# Predictors of health-related quality of life after cardiac surgery: a systematic review

**DOI:** 10.1186/s12955-022-01980-4

**Published:** 2022-05-18

**Authors:** Julie Sanders, Tracey Bowden, Nicholas Woolfe-Loftus, Mandeep Sekhon, Leanne M. Aitken

**Affiliations:** 1 St Bartholomew’s Hospital, Barts Health NHS Trust, West Smithfield, London, EC1A 7DN UK; 2 William Harvey Research Institute, Charterhouse Square, Queen Mary University of London, London, EC1M 6BQ UK; 3 School of Health Sciences, City, University of London, London, EC1V 0HB UK; 4 School of Nursing and Midwifery, Griffith University, Nathan, QLD 4111 Australia

**Keywords:** Health-related quality of live, Predictors, Cardiac surgery, Quality of life, Patient reported outcome

## Abstract

**Background:**

Health-related quality of life (HRQoL) is important in determining surgical success, particularly from the patients’ perspective.

**Aims:**

To identify predictors for HRQoL outcome after cardiac surgery in order to identify potentially modifiable factors where interventions to improve patient outcomes could be targeted.

**Methods:**

Electronic databases (including MEDLINE, CINAHL, Embase) were searched between January 2001 and December 2020 for studies determining predictors of HRQoL (using a recognised and validated tool) in adult patients undergoing cardiac surgery. Data extraction and quality assessments were undertaken and data was summarised using descriptive statistics and narrative synthesis, as appropriate.

**Results:**

Overall, 3924 papers were screened with 41 papers included in the review. Considerable methodological heterogeneity between studies was observed. Most were single-centre (75.6%) prospective observational studies (73.2%) conducted in patients undergoing coronary artery bypass graft (CABG) (n = 51.2%) using a version of the SF-36 (n = 63.4%). Overall, 103 independent predictors (62 pre-operative, five intra-operative and 36 post-operative) were identified, where 34 (33.0%) were reported in more than one study. Potential pre-operative modifiable predictors include alcohol use, BMI/weight, depression, pre-operative quality of life and smoking while in the post-operative period pain and strategies to reduce post-operative complications and intensive care and hospital length of stay are potential therapeutic targets.

**Conclusion:**

Despite a lack of consistency across studies, several potentially modifiable predictors were identified that could be targeted in interventions to improve patient or treatment outcomes. This may contribute to delivering more person-centred care involving shared decision-making to improve patient HRQoL after cardiac surgery.

**Supplementary Information:**

The online version contains supplementary material available at 10.1186/s12955-022-01980-4.

## Introduction

High quality surgical care should include mortality, morbidity and patient-centred outcome measurement [1]. However, patient reported outcomes (PRO) are rarely recorded. Even in research contexts, PROs have only been reported in 29% of cardiac surgery trials [2], despite the fact that those experiencing post-operative complications have worse quality of life [3], which can last three years after surgery [4].

Despite clinicians previously considering health-related quality of life (HRQoL) less important that clinical measures [5], globally health ministers have stated the need to invest in measures that matter most to people [6]. HRQoL measurement allows a holistic, patient-centred perspective of recovery and it is becoming increasingly recognised that HRQoL is important in determining surgical success both from the patients [7] and health-care commissioners [8] perspective.

Factors that predict cardiac surgery mortality do not predict post-operative HRQoL outcome [9]. Thus, an understanding of the factors that do predict HRQoL would be useful to inform patients of the implications of surgery and interventions to improve potentially modifiable predictors could be implemented. Certainly in the UK, HRQoL, and factors associated with it, was identified as the top ten research priority for adult cardiac surgery research [10]. We therefore undertook a literature review to ascertain the predictors of HRQoL after cardiac surgery, to identify potentially modifiable predictors that could be targeted for intervention.

## Methods

### Protocol and registration

This review was registered on PROSPERO, an international prospective register of systematic review (February 2019, reference CRD42019120080) and conducted in accordance with the Preferred Reporting Items for Systematic reviews and Meta-Analyses (PRISMA) guidelines [11].

### Eligibility criteria

All studies that undertook multivariable analysis to identify independent predictors of HRQoL after cardiac surgery were eligible for inclusion. The detailed inclusion and exclusion criteria are detailed in Table 1.

**Table 1:** Inclusion and exclusion criteria

Inclusion criteria	Exclusion criteria
Adult patients (≥ 18 years of age) Primary research English language Published 2001–2020 Patients undergoing cardiac surgical procedures	Surgical ablation procedures in isolation Ventricular Assist Device (VAD) procedures Studies that did not include multivariable analysis of predictors of HRQoL only Congenital heart disease Heart transplantation Transcatheter aortic valve implantation Descriptive exploration of interventions such as cardiac rehabilitation Studies that did not use a validated quality of life instrument Comparison of quality of life in patients who underwent cardiac surgery with those who received percutaneous coronary intervention

### Information sources, search strategy and study selection

A search of MEDLINE, Cumulated Index of Nursing and Allied Health Literature (CINAHL), Embase, Cochrane Library and clinicaltrials.gov (www.clinicaltrials.gov) was undertaken for relevant papers in English between January 2001 and December 2020. Search terms included cardiac surgery OR Cardiac Surgical Procedures AND quality of life OR outcome assessment and were adapted for each database (Additional file 1). Two authors screened the title and abstracts of all citations for suitability against the inclusion and exclusion criteria (Table 1). The reference lists of any identified systematic reviews were also screened for eligible papers.

### Data collection and syntheses (data items and data collection process)

Data were extracted by two authors into a standardised proforma with disagreements resolved through discussion until consensus was achieved. Data extraction included author, country, year, study design, type of surgery, sample size, HRQoL tool used including the time-points where HRQoL was measured, and the independent predictors of HRQoL.

### Risk of bias and quality assessment

All included papers were quality reviewed using an adapted Critical Appraisal Skills Programme (CASP) template for cohort studies (https://casp-uk.net/wp-content/uploads/2018/01/CASP-Clinical-Prediction-Rule-Checklist_2018.pdf). Initial papers were reviewed independently by two authors to ensure consistency and subsequent papers were reviewed by two of four authors with additional random checks undertaken at the end to be assured of continued assurance. A risk of bias graph was generated. Studies were not excluded on the basis of the quality assessment.

### Analysis

Following data extraction, results were summarised using descriptive statistics, tables and narrative synthesis, as appropriate. Interpretation of the analysis was discussed and agreed by all members of the authorship team. Meta-analysis was not possible due to the heterogeneity of studies.

## Results

### Study selection

**Fig. 1:**
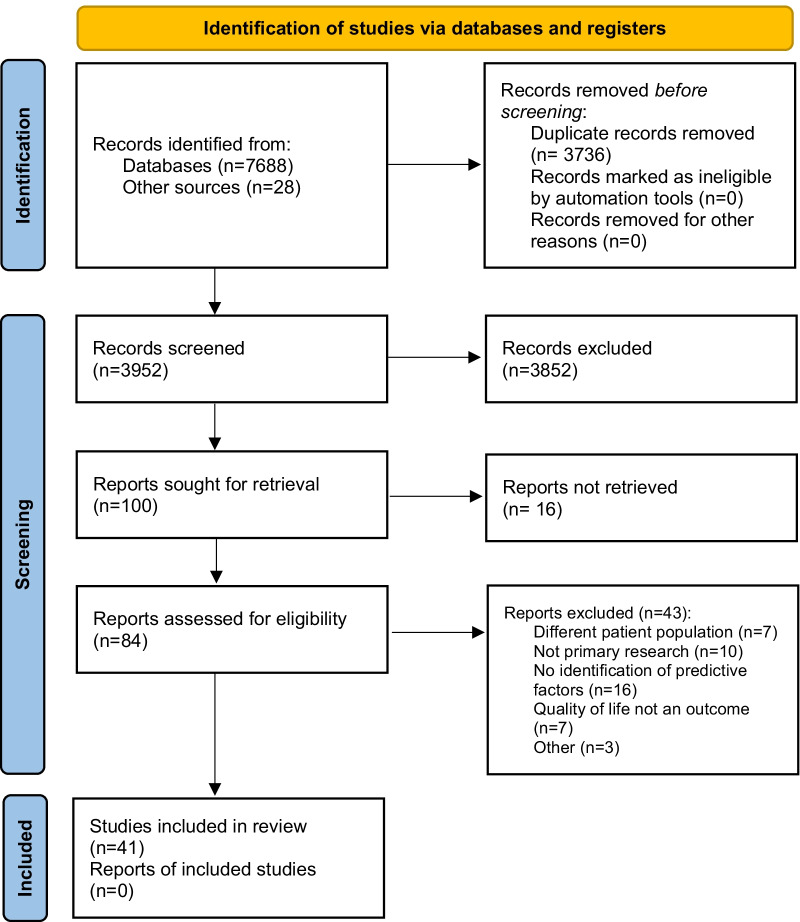
PRISMA flow chart

A total of 3924 papers were identified for possible inclusion (Fig. 1) with 100 papers undergoing independent full-text assessment. This resulted in 41 papers being included for data synthesis.

### Study characteristics

Thirty-two studies were conducted in Europe (two of which were in the UK), four each in Australia the USA, and one in Canada (Table 2). The vast majority were single centre (n = 31) with seven studies conducted in two centres and three studies conducted in multiple centres. Most were prospective observational studies (n = 30) on patients undergoing coronary artery bypass graft (CABG) (n = 21), CABG and/or valve surgery (n = 10), valve only (n = 1) or other combinations of cardiac surgery (n = 9), with sample sizes in the HRQoL analysis ranging from 34 to 8676. The most commonly used tools were versions of the SF-36 (n = 26) and the Nottingham Health Profile (NHP) (n = 8).

**Table 2:** Study characteristics (n = 41)

Study (Author, year, country)	Study design (including (number of sites))	Patients (population (type of surgery) and sample size)			HRQoL tool used and time-point of predictive model (months unless otherwise stated)
		Type of surgery	Sample size		
			Participation rate of eligible persons	Completed follow-up: total cohort	
Myles 2001 [12] Australia	Pre-op post-op (1)	CABG, valve, combined, other	120/125 (96%)	108/120 (90%)	SF-36 (3)
Baldassarre 2002 [15] Canada	Prospective cohort (1)	Isolated CABG (primary)	34/64 (53%)	30/34 (88%)	SF-36 (3)
Falcoz 2003 [34] France	Prospective cohort (1)	CABG, valve, combined, other (elective)	293/452 (65%)	264/293 (90%)	SF-36 (12)
Herlitz 2003 [57] Sweden	Prospective cohort (2)	Isolated CABG (primary)	1225/2000 (61%)	976/2000 (49%)	NHP (10 years)
Schelling 2003 [58] Germany	Prospective cohort (1)	CABG, valve, combined	223/387 (58%)	148/223 (66%)	SF-36 (6)
Baberg 2004 [17] Germany	Prospective and retrospective cohort (1)	AVR ± MVR	201/414 (47%)	201/414 (49%)	SF-36 (3 years)^a^
Jarvinen 2004 [28] Finland	Prospective cohort (1)	Isolated CABG	501/1128 (44%)	458/501 (91%)	SF-36 (12)
Rumsfeld 2004 [18] America	Prospective cohort (14)	Isolated CABG (primary)	2480/3956 (63%)	1973/2480 (80%)	SF-36 (6)
Al-Ruzzeh 2005 [19] UK	Cross-sectional (1)	Isolated CABG (primary)	437/463 (94%)	NA	SF-36 (12)^a^
Herlitz 2005 [59] Sweden	Prospective cohort (1)	Isolated CABG (primary)	1225/2000 (61%)	637/1225 (52%)	NHP (10 years)^b^
Peric 2005 [60] Serbia and Montenegro	Prospective cohort (1)	Elective CABG	243 (no mention of consent/refusal rate)	226/243 (93%)	NHP (6)
Le Grande 2006 [20] Australia	Pre-op post-op (1)	Elective CABG	182/407 (45%)	117/182 (64%)	SF-36 (2, 6)
Myles 2006 [4] Australia	Pre-op post-op (1)	CABG, valve, combined, other	108/120 (90%)	93/108 (86%)	SF-36 (3, 3 years)
Noyez 2006 [61] Netherlands	Retrospective cohort (1)	Isolated CABG	428/428 (100%)	428/428 (100%)	EQ-5D (12)
Panagopoulou2006 [62] Greece	Prospective cohort (1)	Elective CABG	157/256 (61%)	1mo:117/157 (75%) 6mo:104/157 (66%)	MNHD-Q (1, 6)
Dunning 2008 [63] England	Prospective cohort (1)	Isolated CABG	911/1180 (77%)	621/911 (68%)	EQ-5D (10)^a^
El Baz 2008 [32] Netherlands	Prospective observational (2)	CABG	198/256 (73%)	168/198 (85%)	SF-36 (6)
Jokinen 2008 [48] Finland	Prospective observational cohort (1)	CABG, valve, combined, other	91/98 (93%)	46/91 (51%)	NHP (15, 8.2 years)^b^
Peric 2008 [64] Serbia	Pre-op post-op (1)	Isolated CABG	Not reported	192/208 (92%)	NHP (6)
Deaton 2009 [31] USA	Prospective cohort (2)	Isolated CABG	317/442 (72%)	270/317 (85%)	SF-36 (3)^a^
Herlitz 2009 [65] Sweden	Prospective cohort (2)	Isolated CABG (primary)	Not reported 2000 screened	639/2000 (32%)	NHP (15 years)
Maisano 2009 [66] Italy (implied by authorship, not stated)	Retrospective cohort with prospective assessment of HRQoL (1) (implied)	Mitral valve surgery (± AVR, ± TV surgery, ± CABG)	225/225 (100%)	220/225 (98%)	MLHF (3 years)^a^
Rantanen 2009 [67] Finland	Prospective cohort (1)	Elective CABG	1 mo:274/367 (75%) 6 mo:244/271 (90%) 12 mo:236/266 (89%)	1 mo:270/274 (99%) 6 mo:240/244 (98%) 12 mo:235/236 (100%)	15D (1, 6, 12)
Juergens 2010 [30] Germany	Prospective cohort (1)	Elective CABG, valve, combined	56/85 (65%)	42/65 (75%)	SF-12 (3)
Peric 2010 [68] Serbia	Prospective pre-op post-op (1)	Elective CABG	243/243 (100%)	226/243 (93%)	NHP (6)
Grady 2011 [21] America	Prospective cohort (1)	CABG, valve, maze, combined	840/2524 (33%)	0 mo:173/840 (21%) 6 mo:177/840 (21%) 12 mo:174/840 (21%) 24 mo:129/840 (15%) 36 mo:69/840 (8%) Total:816/840 (97%)	SF-36 (3, 6, 12, 2 years, 3 years)
Vainiola 2013 [47] Finland	Prospective cohort (1)	CABG, valve, combined, aortic, other	785/980 (80%)	571/785 (73%)	15-D (6)
Kurfirst 2014 [14] Czech republic	Prospective cohort (1)	CABG, valve, combined (elective)	310 eligible	260/310 (84%)	SF-36 (12)
Humphreys 2016 [33] Australia	Prospective cohort (1)	Elective CABG	180 agreed to participate. No further details	173/180 (96%)	SF-36 (6)
Patron 2016 [22] Italy	Pre-op post-op (1)	CABG, valve, combined (primary, elective)	92/92 (100%)	75/92 (82%)	SF-12 (12)
Bjornnes 2017 [69] Norway	Secondary analysis of RCT (2)	CABG, valve, combined	416/525 (79%)	349/416 (84%)	15D (2 weeks, 3,6,12)
Norkiene 2018 [13] Lithuania	Prospective cohort (1)	CABG, valve, combined	210/210 (100%) No further details	105/210 (50%)	SF-36 (12)
Bishawi 2018 [70] America	Secondary analysis of RCT (18)	Isolated CABG (urgent or elective)	2203/3670 (60%)	1770/2203 (80%)	SAQVR-36 (12)
Grand 2018 [23] France	Prospective cohort (1)	CABG, valve, combined (elective)	495/548 (90%)	326/495 (66%)	SF-36 (6)
Coelho 2019 [24] Portugal	Prospective cohort (1) (implied)	CABG, valve, combined (elective)	Not stated	384/430 (89%)	SF-36 (12)
Blokzijl 2019 [25] Netherlands	Retrospective cohort multicentre (3)	Elective CABG	2606/8643 (30%)	2606/8643 (30%)	SF-36 or SF12 (10–14)
Joskowiak 2019 [26] Germany	Prospective cohort (1)	CABG, valve, combined, aortic, other, redo (elective)	164 consented but does not state number who were eligible and screened	164/164 (100%)	SF-36 (12)
Perrotti 2019 [27] France	Prospective cohort (1)	Isolated CABG (elective)	272/272 (100%)	118/272 (43%)	SF-36 (10 years)
Kube 2020 [35] Germany	Prospective cohort (2)	CABG, valve, combined (elective)	70/110 (64%)	53/70 (76%)	SF-12 (6)
Rijnhart-de Jong 2020 [29]	Prospective cohort (1)	Non-salvage cardiac surgery	1544/1773 (87%)	874/1544 (57%)	SF-36 (12)
Schaal 2020 [71] Germany	Prospective cohort (1)	CABG, valve, combined, aortic,	8676/14043 (62%)	8676/14043 (62%)	NHP (6)

^a^No preoperative HRQoL assessment performed; ^b^ preoperative HRQoL assessment carried out in some, but not all patients

AVR: aortic valve replacement; CABG: Coronary Artery Bypass Surgery; EQ-5D: EuroQol- 5 Dimension; HRQoL: Health-related Quality of Life; MLHF: Minnesota Living with Heart Failure questionnaire; MNHD-Q: MacNew Heart Disease Quality of Life Questionnaire; MVR: mitral valve replacement; NHP: Nottingham Health Profile; PF: physical function; SAQ: Seattle Angina Questionnaire; SF-12: 12 item short form health survey; SF-36: 36 item short form health survey; TV: tricuspid valve; VR-36: Veteran’s Rand (version of SF36)

In most studies HRQoL was measured pre-operatively (n = 35) in addition to at least one post-operative assessment (Table 2), usually within six months of surgery (n = 20) with twenty-four studies assessing outcome at one year or beyond (some studies assessed at more than one time-point). HRQoL was not the primary outcome in all studies.

### Risk of bias

**Fig. 2:**
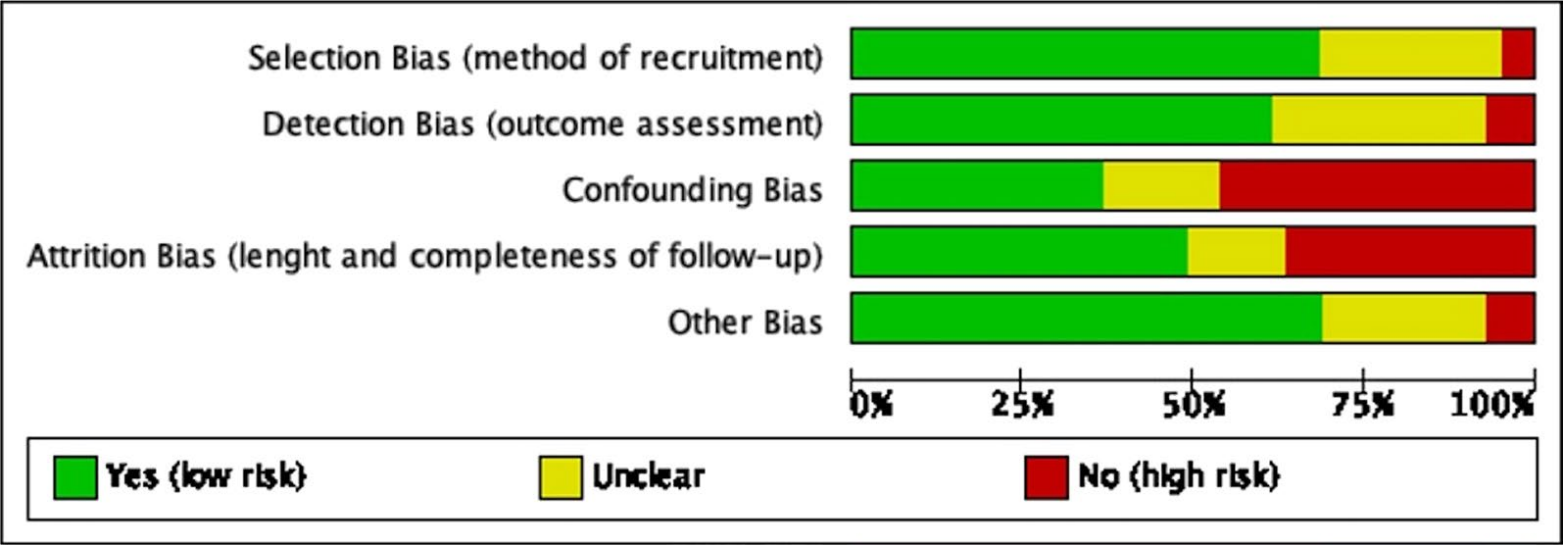
Risk of bias (summary, risk of bias item presented as percentages across all included studies)

**Fig. 3:**
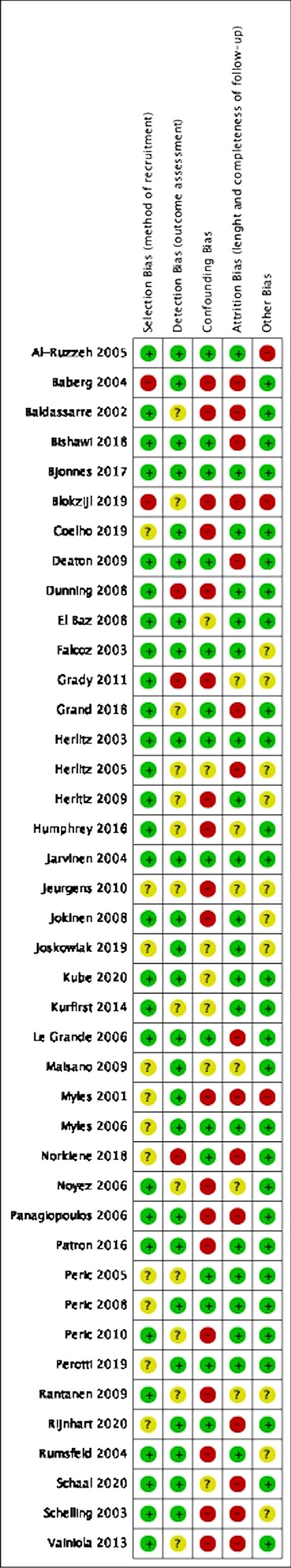
Risk of bias for individual studies

Figures 2 and 3 demonstrate the variable risk of bias across studies and also in considering studies individually.

### Independent predictors of HRQoL

The independent predictors associated with HRQoL by operative and follow-up time-frame are detailed in Table 3 (and by study are included in are detailed in Additional file 1: Table S1). Overall, variables that were examined were predominantly focused on the clinical condition and experience of patients across the pre-, intra- and post-operative course. Of note, few demographic, social or psychological factors were incorporated into the analysis. Despite 26 studies (63.4%) using a version of the SF-36, how it was implemented and categorised to determine predictors varied across studies. For example, four studies explored predictors in relation to the overall score [4, 12–14], 13 explored the physical component (PCS) and mental component score (MCS) separately [15–27], four studies explored one domain [28–31] two studies explored predictors in all SF-36 domains [32, 33] while only Falcoz and colleagues explored both PCS and MCS and all domains [34]. Furthermore, Kube and colleagues used an abbreviated form of the SF-36, the SF-12, to measure physical and psychological quality of life [35].

**Table 3:** Independent variables by operative and follow-up time period

	Independent predictors of HRQoL between 1 and 6 months follow-up	Independent predictors of HRQoL between 1 year and 3.5 years follow-up	Independent predictors of HRQoL at 8.2 years and beyond
Pre-operative variables	Age [15, 18, 33, 35, 71] Alcohol use [33] ASA score [16, 23] Angina [23, 32, 71] BMI [31] Cerebrovascular disease [33, 34, 70] Charlton Comorbidity Index [31] Chronic Heart Failure [33] Chronic neurological disease [18] COPD [18] Delirium [33] Depression [33] Diabetes mellitus [47, 64, 68] Ejection fraction [18, 64] Ethnicity (aboriginal) [33] Everyday functioning questionnaire [20] EuroSCORE [32, 60] FEV1 [18] Gender [64, 68] Gender:Male [47, 71] Gender:Female [31] Geriatric depression scale [31] Health behaviours [33] History of renal insufficiency [32] Hypertension [18, 33] Illness perception questionnaire [30] Living alone [31, 71] NYHA class [18, 20, 32, 71] Peripheral vascular disease [18] Previous cardiac surgery [20] Pre-op 15-D [47] Pre-op expectations (IPQ-E) [35] Pre-op MacNew score [62] Pre-op MCS [18] Pre-op PCS [18] Pre-op SF-12 physical QoL [35] Pre-op SF-12 psychological Qo [35] Profile of mood state vigor-activity [20] Profile of mood state fatigue-inertia [20] Psychiatric disease [18] Psychological distress [62] QoR-40 [12] Serum creatinine [18] Smoking [18, 32, 68] Stress symptom score [16] Work:Not in workforce [20, 71] Work manual occupation [20]	Atrial fibrillation [66] Age [21, 24, 26, 66, 67, 69] Angina class [34] Back/neck problems [69] BMI [21] Chronic Heart Failure [19, 21] COPD [21, 34, 70] Cerebrovascular accident [70] Depression [22, 69, 70] Diabetes Mellitus [17, 29, 66, 70] Education [22, 34, 69] Ejection Fraction [25, 34] Ethnicity (non-white) [21] EuroSCORE [22, 66] Gastrointestinal tract problems [19] Gender [24] Gender:Female [21, 29] Gender/marital status interaction [69] Hypertension [21] Infection [29] MI [21] Mobility [61] Neurological disease [26] NYHA class [17, 21, 34, 66] Other diseases [67] Pain intensity [69] Parsonnet score [34] PCI < 1 year [29] Peripheral or cerebral vascular Disease [34] Peripheral Vascular Disease [19, 21] Pre-op MCS [13, 14, 24–26] Pre-op PCS [13, 14, 22, 24–26, 29] Pre-op SAQ [70] Pre-op SF-36 [4] Pre-op VAS [61] Pre-op VR36 [70] Presence coronary artery disease [21] Pulmonary disease [25] QoR-40 [4] Redo surgery [21] Renal disease [25] Segment wall motion (abnormal) [34] Serum creatinine [66] Type D personality [19] Type valvular heart disease [17]	Age [57, 59, 65] Angina [27] COPD [59] COPD/asthma [63] CCSC [63] Diabetes [27, 48, 59, 63, 65] Duration of cardiac symptoms pre-op > 120 days [48] Dyspnea [27] Gender:Female [63, 65] Height [59] Hypertension [57, 59] NYHA class [57, 65] Obesity [59, 65] Pre-op Nottingham Health Profile [57] Pre-op inferior QoL [65] Protective use of statin [48] Peripheral Vascular Disease [63] Redo surgery [63] Smoking [63]
Intra-operative variables	Cardiopulmonary bypass duration [23] Higher mean pulmonary pressure [20] CABG procedure [71]	On cardiopulmonary bypass [34] Prosthetic valve type [17] CABG procedure [26]	Inotropic drugs at time of surgery [65]
Post-operative variables	Aid from network members [67] Complications [64, 68] Dobutamine [23] Length of hospital stay [32] MI [67] Prolonged LOS [31] New cardiac arrhythmia [20] No of categories of traumatic memory [16] Other diseases [67] Pain:severe and unbearable [47] Physical exertion causing symptoms [67] Post-op expectations (IPQ-E) [35] Post-op SF-12 physical QoL [35] Post-op SF-12 psychological QoL [35] QoL at 1 month (15D) [62, 67] Readmission to hospital within 6 weeks [32] Reexploration [32] Renal replacement for acute renal failure [23] Restlessness during ICU treatment [47] Sternal resuturing [32] Symptoms on mild exertion or at rest [67] Ventilation > 48 h [23]	Infective complications [19] Ejection fraction at follow-up [66] Hospital LOS [4, 24] ICU LOS [24] Mitral regurgitation at follow-up echo [66] Perioperative MI [28] QoL at 1 month (15D) [67] Quality of life at 3 months [4] Poor QOR-40 at 1 month [4] Physical exertion causing symptoms [67] Post-operative rhythm [17] Sternal complications [17] Symptoms on mild exertion or at rest [67] Systolic pulmonary artery pressure at follow-up [66]	High pain score at 15 months [48] ICU time [65] ICU 2 days [63] ICU > 3 days [48] Length ventilator time [59] Low energy score at 15 months [48]

BMI: Body Mass Index; CABG: Coronary Artery Bypass Graft; CCSC: Canadian Cardiovascular Society; COPD: Chronic obstructive pulmonary disease; FEV1: Forced Expiratory Volume; ICU: Intensive Care Unit; LOS: Length of Stay; MCS: mental component score; MI: Myocardial Infarction; NYHA: New York Heart Association classification; PCI: Percutaneous Coronary Intervention; PCS: Physical Component Score; QoL: Quality of Life; SAQ: Seattle Angina Questionnaire; VAS: Visual Analogue Scale; VR36: Veteran’s Ran

Due to the variation in analysis and reporting across the studies, the independent predictors identified were grouped by operative and follow-up time-frame (Table 3). In total, 103 independent predictors (62 pre-operative, 5 intra-operative and 36 post-operative) were identified. Of those 103 variables 34 (33.0%) were identified as significant in more than one study and almost all of those (n = 33 (97.1%)) were also found to be non-significant in other studies (non-significant variable data detailed in Additional file 2: Table S2). Variables found to be predictive at all three time-points were age, angina, chronic obstructive pulmonary disease (COPD), diabetes, gender, hypertension and NYHA class and peripheral vascular disease.

### Potentially modifiable predictors

Of the 62 pre-operative variables identified as independent predictors for HRQoL outcome those that are potentially modifiable pre-surgery include alcohol use, body mass index (BMI)/weight, depression, pre-operative quality of life and smoking (Table 3).

Similarly, in the post-operative period independent predictors with the potential to be modified to improve HRQoL outcome were pain, traumatic memories and restlessness in the intensive care unit (ICU). Furthermore, general strategies to reduce post-operative complications (including infection, myocardial infarction, arrythmias and readmission) and shorten ICU and hospital length of stay are also identified as potential targets to improve post-surgical HRQoL (Table 3).

## Discussion

The inclusion, measurement and use of HRQoL and PRO in routine cardiac surgery practice is lacking. Healthcare organisations need to work with patients to deliver more person-centred care, sharing decision-making, to meaningfully improve care outcomes [36]. The ‘holy grail’ of prognostic factor research is to improve patient outcomes by providing a personalised approach to healthcare and risk prediction and how such factors could be used to improve patient or treatment outcomes [37]. Thus, we sought to identify known predictors for HRQoL outcome after cardiac surgery, specifically to focus on modifiable factors where interventions to improve patient or treatment outcomes could be targeted. We identified 41 studies, which were predominantly European-based single-centre prospective observational studies conducted in CABG patients. Certainly, recognition of the non-modifiable predictors found to be particularly impactful both on short and longer-term HRQoL (age, angina, COPD, diabetes, gender, hypertension and NYHA class and peripheral vascular disease) may assist in identifying high risk patients and the identification of interventions and associated resources that might then be directed to assisting these patients to recover. In terms of potential modifiable predictors, pre-operative factors include alcohol use, smoking, BMI/weight depression, and pre-operative quality of life, while ongoing pain management, prevention of post-operative complications and general strategies to reduce ICU and hospital length of stay could also be beneficial.

Individually focused lifestyle and therapeutic interventions have shown effectiveness in weight and BMI reduction [38], decreasing alcohol consumption [39], psychological preparation (including depression and anxiety) [40] and smoking cessation [41]. Given that BMI [42], alcohol use [43], depression and anxiety [44] and smoking [45] have also been identified to be associated with many in-hospital post-operative complications, strategies to encourage their reduction are likely to have beneficial impacts on improving overall morbidity and general recovery. As yet, interventions specifically targeting pre-operative HRQoL do not exist. While most tools combine physical, mental and social wellbeing traditionally greater emphasis clinically has placed on physical health. Nonetheless, the importance of psychological readiness and inclusion of social support and anxiety reduction in prehabilitation programmes is now recognised as part of cardiac surgery enhanced recovery [46]. Furthermore, we found that severe pain during the ICU stay was an independent predictor of HRQoL at six months [47], while high pain scores at 15 months were predictive of HRQoL eight years after surgery in elderly patients [48]. Since up to 10% of cardiac surgery patients develop severe chronic post-surgical pain [49], with predictors of chronic pain including early severe pain [50] personalised effective pain management is vital. Current recommendations suggest the use of multimodal opioid-sparing pain management alongside the use of a pain assessment tool to ensure the lowest opioid dose [46].

Certainly, future work requires more methodologically robust studies, including large multi-site studies with appropriate control of confounding factors. However, generally a greater emphasis on HRQoL as an outcome measure after cardiac surgery, both clinically and in research, is needed. Although HRQoL has been previously undervalued by clinicians [5], the landscape is changing with the importance of HRQoL now recognised in cardiac surgery clinical guidelines [51], the enhanced recovery recommendations [46], the cardiac surgery core outcome dataset [52] and that PROs are included in the Swedish national registers [53] and emerging in the USA STS National Database [54]. Similarly, HRQoL is reported as a top research priority in cardiac surgery, both in the UK [10] and in the USA [55]. Therefore, our review is timely, in that it collates the available evidence on predictors of HRQoL, highlights potential modifiable factors on which interventions could be based in improve patient outcome and emphasises where greater research quality in prognosis factor research should reside in this area.

### Strengths and limitations

Despite the methodological robustness of this review, there are three main limitations. Firstly, the methodological heterogeneity of the included studies restricts the ability to make strong conclusions or undertake a meta-analysis. Our review reflects that despite the considerable growth in prognosis research, the quality is often sub-standard [56]. Secondly, although only English language publications were included, studies from around the World have been included, providing a relatively wide base of evidence. Finally, included studies were limited to those published from 2001. A balance was struck between including all evidence and ensuring the results of this review were clinically appropriate outcome predictors for the current time. A period of 20-years was deemed sufficient to address the balance needed.

In conclusion, despite a lack of consistency across studies, several potentially modifiable predictors on which interventions to improve patient HRQoL outcomes could be targeted were identified. While this review has robustly collated the current best prognosis factor evidence relating to predictors of HRQoL after cardiac surgery, there is still a need for large multi-site studies, with appropriate control of confounding factors, to examine the role of these factors in affecting HRQoL outcome. Now that considerably more emphasis is placed on the importance of HRQoL and PROs after cardiac surgery, the hope is that this will contribute to delivering more person-centred care involving shared decision-making to improve patient short- and longer-term recovery.

## Implications for practice

- Cardiac surgery and enhanced recovery guidelines highlight the importance of HRQoL
- Pre-operative lifestyle and therapeutic interventions relating to weight, alcohol use, psychological preparation and smoking cessation may improve HRQoL
- Reducing chronic post-operative pain, in-hospital complications and length of hospital stay could also improve HRQoL.
- More person-centred care, including HRQoL and shared decision-making, is needed to improve patient short- and longer-term recovery.

## Supplementary Information

**Additional file 1: **Search strategy.

## Data Availability

Not applicable.

## Abbreviations

BMI: Body mass index
CABG: Coronary artery bypass graft
CASP: Critical Appraisal Skills Programme
COPD: Chronic obstructive pulmonary disease
HRQoL: Health-related quality of life
ICU: Intensive care unit
MCS: Mental component score
NYHA: New York Heart Association
PCS: Physical component score
PRISMA: Preferred Reporting Items for Systematic reviews and Meta-Analyses
PRO: Patient reported outcomes
PROSPERO: International Prospective Register of Systematic Reviews
SF-36: Short-Form 36
