# Supplementary material for: Predictors of health-related quality of life after cardiac surgery: a systematic review
Source: Health Qual Life Outcomes. 2022 May 18;20:79. doi: 10.1186/s12955-022-01980-4 (PMC9118761; doi:10.1186/s12955-022-01980-4)
Supplement: Supplementary file 1 — Additional file 1: Search strategy. [file 12955_2022_1980_MOESM1_ESM.docx]

**SUPPLEMENTARY MATERIAL**

**Supplementary material 1:** Search strategy

**Cochrane**
("cardiac surgery" OR Cardiac Surgical Procedures [mh:noexp]) AND ("quality of life" OR Quality of Life [mh:noexp] OR "outcome assessment" OR "Outcome Assessment (Health Care)" [mh:noexp])

Limited to Jan 2001 – 2020, English, no exploding of MeSH

**Medline**

("cardiac surgery" OR Cardiac Surgical Procedures [mh:noexp]) AND ("quality of life" OR Quality of Life [mh:noexp] OR "outcome assessment" OR "Outcome Assessment (Health Care)" [mh:noexp])

Limited to Jan 2001 – 2020, English, no exploding of MeSH

**Embase**

('cardiac surgery' OR 'heart surgery'/exp/mj OR 'heart surgery'/mj) AND ('quality of life'/exp/mj OR 'quality of life'/mj OR 'outcome assessment'/exp/mj OR 'outcome assessment'/mj) AND [humans]/lim AND [english]/lim AND [embase]/lim AND [2001-2020]/py

Limited to English, 2001 – 2020, ‘major focus’ only, humans.

**CINAHL**

("Cardiac Surgery" OR (MH "Heart Surgery")) AND ((MH "Quality of Life") OR (MH "Outcome Assessment"))

Limited to 2001 – 2020, English.

**Clinicaltrials.gov**

("cardiac surgery" OR “Heart Surgery”) AND ("quality of life" OR "Outcome assessment")

**Supplementary Table 1:** Results of individual studies, detailing independent variables associated with HRQoL

| **Study**  (Author, year) | **Post-operative HRQoL follow-up time points** (months, unless otherwise stated) | **Independent predictors of HRQoL** |
| --- | --- | --- |
| Myles 2001^1^ | 3 | **SF36 total**: QoR-40 |
| Baldassarre 2002^2^ | 3 | **SF36 PCS:** Age  **SF36 MCS:** Age |
| Falcoz 2003^3^ | 12 | **SF36 PCS:** Angina class  **SF36 MCS:** Parsonnet score, NYHA class  **SF36 Physical function:** NYHA class, Angina class, segmental wall motion  **SF36 Physical role:** COPD, CVD or PVD  **SF36 Bodily pain:** Angina class, NYHA class  **SF36 Mental health:** COPD presence, NYHA class  **SF36 Social role:** LV **e**jection fraction, beating heart – absence, NYHA class  **SF36 General health:** Education level, angina class, NYHA class |
| Herlitz 2003^4^ | 10yrs | **NHP:** Preop QOL, age, NYHA class, hypertension |
| Schelling 2003^5^ | 6 | **SF36 PCS:** Stress symptom score, ASA score, number of categories of traumatic memory  **SF36 MCS:** Stress symptom score |
| Baberg 2004^6^ | Mean 42.5 ± 23.1 | **SF36 PCS:** NYHA class, sternal complications, Diabetes mellitus, prosthetic valve type  **SF36 MCS:** NYHA class, sternal complications, postoperative rhythm, type of valvular heart disease |
| Jarvinen 2004^7^ | 12 | **SF36 General health:** perioperative AMI |
| Rumsfeld 2004^8^ | 6 | **SF36 PCS:** Chronic neurologic disease, PVD, COPD, hypertension, LV EF, Forced Expiratory Volume (FEV)1, current smoking, preoperative SF36 MCS, serum creatinine  **SF36 MCS:** Psychiatric disease, COPD, current smoker, elevated NYHA, preoperative PCS, age |
| Al-Ruzzeh 2005^9^ | 12 | **SF36 PCS**: Gastro-intestinal problems, CHF, Type D personality  **SF36 MCS**: PVD, infective complication, Type D personality |
| Herlitz 2005^10^ | 10yrs | **NHP total:** Age, hypertension, diabetes mellitus, COPD on medication, obesity, height, length of ventilator time, preoperative QOL |
| Le Grande 2006^11^ | 2 and 6 | **SF36 PCS**: NYHA score, new cardiac arrhythmia, mean pulmonary pressure, work status, POMS vigor-activity, fatigue-inertia  **SF36 MCS**: POMS depression-dejection, manual occupation, Everyday Functioning Questionnaire concentration, previous cardiac surgery |
| Myles 2006^12^ | 3yrs | **SF36:** Duration of hospital stay, pre-op SF-36, QoL at 3 months, pre-op QoR-40, QoR at one month |
| Noyez 2006^13^ | 1yr | **EQ-5D:** Pre-mobility level, preoperative current state of health |
| Panagopoulou 2006^14^ | 1 and 6 | **MNHD-Q QOL 1 mth:** Quality of life on admission; preoperative psychological distress  **MNHD-Q QOL 6 mths:** Quality of life at 1 mth; preoperative psychological distress |
| Dunning 2008^15^ | 10yrs | **EQ 5D:** current smoker, Canadian Cardiovascular Society III or IV, redo surgery, ICU LOS, sex, diabetes mellitus, PVD, COPD or asthma |
| El Baz 2008^16^ | 6 | **SF36 Physical function:** EurosSCORE, readmission to hospital within 6 weeks, NYHA class  **SF36 Physical role**: Readmission, hospital LOS  **SF36 Bodily pain**: Smoking, history of renal insufficiency  **SF General health**: Readmission to hospital  **SF36 Social role**: Readmission, hospital LOS  **SF36 Emotional role**: Smoking  **SF36 Mental health**: Reexploration, sternal resuturing, readmission within 6 weeks, angina  **SF36 Vitality**: Reexploration, readmission to hospital within 6 weeks |
| Jokinen 2008^17^ | 8.2yrs | **NHP Energy:** Diabetes, low energy score at 15 months, protective use of statin, treatment in ICU>3days  **NHP Sleep:** High pain score at 15 months  **NHP Pain:** Diabetes mellitus  **NHP Emotional reactions:** Duration of cardiac symptoms pre-op >120 days  **NHP Physical mobility:** Diabetes mellitus, low energy score at 15 months |
| Peric 2008^18^ | 6 | **NHP Physical mobility**: Diabetes mellitus, LV EF  **NHP Social Isolation**: Complications  **NHP** Emotional reactions: LV EF  **NHP** **Sleep**: Complications  **NHP** **Pain**: Gender, complications |
| Deaton 2009^19^ | 3 | **SF36 PCS:** Charlson Co-morbidity Index, Geriatric Depression Scale, sex, living alone, BMI, postoperative LOS |
| Herlitz 2009^20^ | 15yrs | **NHP**: Age, sex, Diabetes mellitus, obesity, ICU LOS, inotropic drugs, pre-op inferior QoL, NYHA class |
| Maisano 2009^21^ | 2.8 ± 1.2yrs | **MLHF**: Preoperative AF, Diabetes mellitus, creatinine level, EuroSCORE, mitral regurgitation grade at follow-up echo, systolic PAP at follow-up, age, preoperative NYHA class, LV EF at follow-up |
| Rantanen 2009^22^ | 6 and 12 | **EQ 15D** (6 months): 15D score at 1 month, physical exertion causes symptoms, symptoms on mild exertion or at rest, experienced AMI, preoperative other disease, aid from network members  **EQ 15D** (12 months): 15D score at 1 month, physical exertion causes symptoms, symptoms on mild exertion or at rest, preoperative other disease, age |
| Juergens 2010^23^ | 3 | **SF12 Physical function: I**llness perception questionnaire – revised (IPQ-R) |
| Peric 2010^24^ | 6 | **NHP Physical mobility**: Diabetes mellitus, complications  **NHP Social isolation**: Smoking, complications  **NHP Sleep**: Complications  **NHP Pain**: Gender, complications |
| Grady 2011^25^ | Over time (0, 3, 6, 12 and 24) | **SF36 PCS**: Prior myocardial infarction, age, BMI  **SF36 MCS**: BMI, heart failure |
| Vainiola 2013^26^ | 6 | **EQ 15D**: Male, diabetes mellitus, baseline 15-D, severe or unbearable pain, restlessness during ICU treatment |
| Kurfirst 2014^27^ | 12 | **SF36 total**: Pre-op PCS, pre-op MCS |
| Humphreys 2016^28^ | 6 | **SF36 Bodily pain**: Alcohol use, depression  **SF36 General health**: Alcohol use, CHF, depression, delirium  **SF36 Vitality**: Alcohol use, depression  **SF36 Social functioning**: Alcohol use, health behaviours, depression  **SF36 Emotional role**: Alcohol use, cardiovascular disease, depression  **SF36 Mental health**: Alcohol use  **SF36 Physical function**: Alcohol use, CHF, hypertension, Aboriginal, age  **SF36 Physical role**: Alcohol use, age |
| Patron 2016^29^ | 12 | **SF36 PCS:** Pre-op PCS, education, EuroSCORE, CES-D  **SF36** **MCS**: Pre-op MCS, CES-D |
| Bjonnes 2017^30^ | 2 weeks, 3, 6, and 12 | **EQ 15D:** Age, sex x marital status, educational status, back/neck problems, depression, pain intensity |
| Norkiene 2018^31^ | 1yr | **SF36 total:** Preoperative PCS, preoperative MCS |
| Bishawi 2018^32^ | 12 | **SAQ-QoL:** COPD, depression, baseline SAQ-QoL  **VR36 PCS**: Diabetes mellitus, baseline VR36 PCS  **VR36 MCS**: CVA, depression, baseline VR36 MCS |
| Grand 2018^33^ | 6 | **SF36 PCS:** ASA physical status, CPB duration, renal replacement for acute renal failure, mechanical ventilation > 48 hours  **SF36 MCS**: Preoperative angina, postoperative dobutamine, renal replacement for acute renal failure, mechanical ventilation > 48 hours |
| Coelho 2019^34^ | 12 | **SF36 PCS:** Preoperative PCS, sex, age, hospital LOS  **SF36 MCS:** Preoperative MCS, ICU LOS |
| Blokzijl 2019^35^ | 10 – 14 | **SF36 PCS:** Baseline PCS, pulmonary disease, LV function, renal disease  **SF36 MCS:** Baseline MCS, LV function |
| Joskowiak 2019^36^ | 3 and 12 | **SF36 PCS:** Baseline PCS, age, pre-operative neurological disease  **SF36 MCS:** Baseline MCS, age, CABG procedure |
| Perrotti 2019^37^ | 10yrs | **SF36 PCS:** Diabetes, dyspnea  **SF36 MCS:** Angina |
| Kube 2020^38^ | 6 | **SF12 – Physical QoL:** pre-surgical Physical QoL, age (p*re-surgery model*); post-surgery Physical QoL, post-surgery expectations (IPQ-E pre-op) (p*ost-surgery model)*  **SF12 – Psychological QoL:** presurgical Psychological QoL, presurgical expectations (IPQ-E pre-op) (p*re-surgery model*); post-surgical Psychological QoL, post-surgical expectations (IPQ-E 7 – 10 days post-op) (p*ost-surgery model*) |
| Rijnhart de Jong 2020^39^ | 1yr | **SF36 PF:** Baseline SF36 PF, diabetes, sex, infection, PCI <1 yr |
| Schaal 2020^40^ | 6 | **NHP:** Gender, age, marital status, occupation, type of cardiac surgery, chest pain, NYHA class |

Note: Peric et al 2005 not included above due to no significant predictors reported

Abbreviations: AMI – Acute Myocardial Infarction; ASA – American Society of Anesthesiologists risk classification; BMI – Body Mass Index; CES-D – Centre for Epidemiological Study of Depression; CHF – Chronic Heart Failure; CVA – Cerebrovascular accident; CVD – cerebral vascular disease; LV EF – Left ventricular ejection fraction; EQ: Euroqol; ICU – Intensive Care Unit; LOS – Length of Stay; MNHD-Q: MacNew Heart Disease Quality of Life Questionnaire; MCS – Mental Component Score; NHP: Nottingham Health Profile; NYHA – New York Heart Association; PCI <1 yr – Percutaneous Coronary Intervention <1 year prior; PCS – Physical Component Score; POMS - Profile of Mood States; PVD – Peripheral Vascular Disease; QoL – Quality of Life; QoR – Quality of Recovery; SAQ Seattle Angina Questionnaire; SF-36: Short Form 36; VR36 – Veteran’s Rand (veteran version of the SF36).

d (veteran version of the SF36); Yrs: years.

**Supplementary Table 2:** Results of individual studies, detailing independent and non-significant variables associated with HRQoL

| **Study**  (Author, year) | **Post-operative HRQoL follow-up time-points where multivariate analysis conducted (months unless other wise stated)** | **Independent predictors of HRQoL (***with data where given)* | **Non-significant variables in multivariate analysis** |
| --- | --- | --- | --- |
| Myles 2001^1^ | 3 | QoR-40 (p<0.003) | Age, gender, marital status, type of surgery, number of grafts, Tu score, pre-bypass cardiac index, cross-clamp time, duration of surgery, post-op cardiac index, time to tracheal extubation, duration of ICU stay, duration of hospital stay, QoR-40 (each domain), respiratory complication, cardiovascular complication, stroke, sepsis acute renal failure. |
| Baldassarre 2002^2^ | 3 | **PCS:** Age: β -0.66 (-1.17 to -0.15), p = 0.01. **MCS**: Age: β 0.69 (0.19 to 1.19), p = 0.01 | **PCS:** Hypertension, previous MI, diabetes, number of diseased vessels, FT at T1, MCS at T1, married, University or college education, Income ≥ $40,000/yr,  **MCS**: Hypertension, previous MI, diabetes, number of diseased vessels, FT at T1, PCS at T1, married, university or college education, income ≥ $40,000/yr |
| Falcoz 2003^3^ | 12 | **PCS:** angina class III-IV (β 2.78 [1.23 - 6.27]). **MCS:** Parsonnet score C-D (β 2.98 [1.02 - 8.69]) NYHA class III-IV (β 2.85 [1.28 - 6.38])  **Physical domain:** NYHA class III-IV (β 2.84 [1.36 - 5.92]), Angina class III - IV (β 2.16 [1.05 - 4.45]), Abnormal segmental wall motion (β 1.91 [1.03 - 3.56]). **Physical role domain:**  COPD presence (β 2.99 [1.14 - 7.87])  CPVD (cerebral or peripheral vascular disease) presence (β 1.98 [1.01 - 3.87]). **Pain domain:**  Angina class III - IV (β 3.32 [1.67 - 6.61]) NYHA class III-IV (β 2.31 [1.20 - 4.46]). **Energy domain** – nil. **Mental domain** – nil. **Mental role domain:** COPD presence (β 3.47 [1.20 - 10.1]) NYHA class III-IV (β 2.07 [1.06 - 4.03])  **Social domain:** Ejection Fraction <0.5 (β 2.82 [1.34 - 5.60 ]), Beating heart - absence (β 2.80 [1.33 - 5.95]), NYHA class III - IV (β 2.74 [1.09 - 7.22]). **General domain:** Level of study - higher (β 3.38 [1.34 - 8.55]), Angina class III - IV (β 2.22 [1.14 - 4.33]), NYHA class III-IV (β 1.97 [1.03 - 3.78]) | Factors assessed for all analyses included:  - sociodemographic (age, sex, family situation, level of study),  - angina pectoris status according to the CCS, dyspnea class according to the NYHA classification (class II was divided into two subgroups: II-mild for patients not troubled by shortness of breath when walking up a slight hill at a normal pace, and II-severe for patients who had to stop for breath when walking up a slight hill at their own pace), ejection fraction, left ventricular wall motion,  - surgical preoperative risk estimation scores (Parsonnet score and EuroSCORE)  - comorbid diseases,  - type of heart operation,  - operative complications. |
| Herlitz 2003^4^ | 10 | **NHP:** Preop QoL: OR 2.5 (1.7 - 3.5), p<0.0002; Age: OR 0.96 (0.94 - 0.98), p = 0.0002; Functional class (NYHA): OR 1.6 (1.2 - 2.2), p = 0.001; Hypertension: OR 0.5 (0.4 - 0.8), p = 0.0005 | Factors tested on univariate analysis to go into multivariate: Pre-op factors (sex, age, NYHA class, previous MI, angina, CHF, high BP, Diabetes, renal dysfunction, cerebrovascular disease, obesity, smoker, previous PTCS, 3 vessel disease, EF<40%); Pre-op QoL, History of valvular disease, intermittent claudication, COPD on medication, length, weight, body surface area, left main and proximal LAD stenosis, Peri-op factors associated with operation (urgency, extra-corporeal circulation time, aortic cross clamp time, number of distal anastomes, IMA grafting, endarterectomy, ICU LOS, ventilator time, need for reoperation, neuro complication, pneumothorax, hydrothorax, SVT, inotropes, circulatory assist devise, serum aspartate amino-transferase max. |
| Schelling 2003^5^ | 6 | **PCS:** stress symptom score (B -0.46, -0.74--0.18, p<0.01), ASA score (B-4.08, -7.84--0.33, p=0.03), No of categories of traumatic memory (B-2.19, -4.36--0.07, p=0.04). **MCS:** stress symptom score (R2 0.52, B=-0.75, -0.93--0.53, residual SD 7.18, p<0.01). | **PCS:** duration of CPB, number of hours spent in ICU. **MCS:** duration of CPB, number of hrs spent in ICU, ASA score, No of categories of traumatic memory |
| Baberg 2004^6^ | mean 42.5 ± 23.1 | **PCS:** NYHA class (beta -0.363, p=0.001); Sternal complications (-0.199, p=0.010); diabetes mellitus (-0.151, p=0.032); prosthetic valve type (-0.148, p=0.039). **MCS:** NYHA class (-0.225, p=0.004); sternal complications (-0.196, p=0.011); postoperative rhythm (-0.188, p=0.16); type of valvular heart disease (-0.164, p=0.031) | **PCS:** age; gender; accompanying illnesses; aortic valve pathology; preoperative peak aortic gradient; ejection fraction; left ventricular diameter; associated procedures; postoperative complications; time operation; cardiac rhythm  **MCS**:age; gender; accompanying illnesses; preoperative peak aortic gradient; ejection fraction; left ventricular diameter; associated procedures; valve prosthesis type; postoperative complications; time since operation; |
| Jarvinen 2004^7^ | 12 | **GHS:** perioperative AMI: (OR 1.78, 1.04-3.07, p<0.05) | **GHS:** age, female, diabetes, EuroSCORE, Urgent or emergent operation, Redo surgery, LMS stenosis, Subnormal EF (<50%), Post-op events - AF, other complication, Ventilatory support >36hr, ICU stay> 3days. |
| Rumsfeld 2004^8^ | 6 | **PCS:** Chronic neurologic disease -3.17(1.35) p 0.019, Peripheral vascular disease -2.28(0.51) p <0.001, Chronic obstructive pulmonary disease -1.76(0.72) p0.015, Hypertension -1.33(0.45) 0.003, Left ventricular ejection fraction -1.24(0.44) p 0.005, FEV1 1.19(0.33) p <0.001, Current smoking -1.16(0.52) p 0.027, Pre op mental component -0.85(0.19)<0.001, Serum creatinine -0.74(0.31) p 0.017. **MCS:** Psychiatric disease -5.77(1.07) p <0.001, COPD -2.04(0.82) p 0.012, Current smoker -1.63(0.63) p 0.010, Elevated NYHA -1.31(0.54) p0.015, Preop physical component summary -0.91(0.29) p0.002, Age 0.83(0.30) p 0.005 | male sex, body surface area, diabetes, cerebrovascular disease, alcohol abuse, liver disease, Canadian cardiovascular society angina class, family history of coronary heart disease, prior heart surgery, surgical priority, MI within 7 days, intravenous nitro-glycerine preoperatively, IABP preoperatively, diuretic use, pulmonary rales, cardiomegaly on chest radiography, resting ST segment depression on ECG, left main stem disease, 3 vessel disease |
| Al-Ruzzeh 2005^9^ | 12 | **PCS scores**: (OR (95%CI) p): Gastrointestinal problems (2.21 (1.33-3.67) p=0.002), CHF (2.01 (1.03-3.92) p=0.04), Type D personality (2.34, (1.51-3.61) p<0.001). **MCS scores**: (OR (95%CI) p): PVD (2.49 (1.11-5.57) p=0.03), infective complication (1.96 (1.2-3.19) p=0.007), Type D personality (5.51 (3.49-8.73) p<0.001) | **PCS:** Diabetes, PVD, Dyspnoea (NYHA III/IV), Pre-op CVA/TIA, BMI>30kg/m2, Infective complications, readmission to ITU.  **MCS:** Age >70, Dyspnoea (NYHA III/IV), Smoking, respiratory problems, CHF, Preop-CVA/TIA, pulmonary complications, Anxiety. |
| Herlitz 2005^10^ | 10 | **NHP total score** (OR 95%CI, p): Age OR 1.03(1.01-1.05) p 0.0007, Hypertension OR 1.3(1.0-1.8) p 0.05, Diabetes OR 1.9(1.1-3.2) p 0.01, COPD on medication OR 6.1(1.4-27.2) p 0.02, Obesity OR 2.2(1.4-3.4) p 0.0007, Height OR 0.97(0.95-0.98) p 0.0001, length of ventilator time OR 1.5(1.1-2.1) p0.006  When inferior QoL before surgery was incorporated into analysis, this was an independent predictor:  NHP OR 3.9(2.7-5.6) p <0.0001, PGWBI OR 5.1(3.5-7.3) p < 0.0001, PAS OR 1.8(1.3-3.7) p 0.001 | **NHP:** female sex, NYHA class, previous MI, angina pectoris, congestive heart failure, renal dysfunction, cerebrovascular disease, claudication, current smoker, previous PTCA, 3 vessel disease, EF<040, angina duration, history of valvular disease, weight, body surface area, left main and proximal left anterior descending stenosis, urgency of operation, ECC time, aortic cross clamp time, number of distal anastomoses, IMA grafting, endarterectomy, time in ICU, need for reoperation, neurological complication, pneumothorax, hydrothorax, supraventricular arrhythmia, prolonged reperfusion in the heart lung machine, need for inotropic drugs, need for circulatory assist device, S-ASAT maximum within 48 hrs |
| Le Grande 2006^11^ | 2 and 6 | **Non-improver of PCS**: Higher NYHA (OR 1.87, 1.18-2.95, p<0.007), new cardiac arrhythmia (OR 2.62, 1.13-6.05, p=0.024), higher mean pulmonary pressure (OR 1.09, 1.01-1.17, p=0.03), not in the workforce (OR 2.38, 1.02-5.56, p=0.45), lower POMS vigor-activity (OR 0.93, 0.87-0.97, p=0.003), higher fatigue-inertia (results not stated in paper). **Non-improver of MCS**: POMS depression-dejection (OR1.08, 1.03-1.13, p=0.002), manual occupation (OR2.66, 1.25-5.65, p=0.011), EFQ (everyday functioning questionnaire concentration (OR1.30, 1.02-1.66, p=0.037), previous cardiac surgery (OR5.17, 1.09-27.74, p=0.044). | No variables associated with change in PCS over time.  **Non-improver of PCS**: AF, CHF, higher BMI, previous MI, female, born outside Australia. **Non-improver of MCS:** presence of AF, having off-pump surgery, previous MI, non-attendance at cardiac rehabilitation, not having a current partner, POMS domains except those significant; EFQ domains (memory, organisation, communication) |
| Myles 2006^12^ | 3 years | Duration of hospital stay (days) (p0.004), pre-op SF-36 (p=0.021), poor QoL at 3 months (p<0.001), pre-op QoR-40 (p=0.005), poor QoR at one month (p=0.003) | Age, female gender, lives with spouse, weight, CABG vs other, pre-op beta blockers, Tu score, cross-clamp time (min), time to tracheal extubation, (hrs), duration of ICU stay (hrs), duration of hospital stay (days), cardiac index (pre-bypass and ICU admission), any post-operative complications, pre-op poor QoL, QoR-40 at 3 days, poor QoR at 3 days. |
| Noyez 2006^13^ | 12 | Pre-mobility>level 1 p=0.008, OR=2.01, 95%CI (1.2–3.7), Preoperative VAS<60 p=0.000, OR=2, 95%CI (2 1.6–3.0) | Age ≥75 years, Sex, Diabetes, Vascular disease, Lung disease, Pre-NYHA, Pre-self-care>level 1, Pre-usual activities>level 1, Pre-pain/discomfort level>1, Pre-anxiety/depression>level 1 |
| Panagopoulou 2006^14^ | 1 and 6 | **QoL 1 mth after surgery:** QoL on admission β = 0.34; p<0.001, preoperative psychological distress: β = -0.22, p<0.01. **QoL 6 mths after surgery:** QoL at 1 mth: β = 0.34, p<0.001, preoperative psychological distress: β = -0.28, p<0.001 | No data or detail provided, but these factors were incorporated: QoL on admission, preoperative distress, preoperative functional status, age, number of diseased vessels, social support |
| Dunning 2008^15^ | 10 | **Poor Outcome by EQ-5D:** Current smoker p=0.000 (OR: 3.978, 95% CI: 2.158-7.333), Canadian Cardiovascular Society III or IV p=0.000 (OR: 4.729, 95% CI: 2.170-10.303)  Redo surgery p=0.001 (OR 6.226, 95% CI: 2.080-18.632), 2 days in ICU p= 0.003 (OR: 2.323, 95% CI: 1.325-4.074), Female sex p=0.016 (OR: 2.088, 95% CI: 1.148-3.800), Any diabetes p=0.020 (OR: 2.524, 95% CI: 1.156 5.510), Peripheral vascular disease p= 0.026 (OR: 2.088, 95% CI: 1.092 3.991), COPD or asthma p=0.049 (OR: 2.263, 95% CI: 1.005-5.097) | **Poor Outcome by EQ-5D:** Presence of hypertension, Endarterectomy, IABP, 3 grafts or fewer, any nitrates or heparin, Left mainstem disease, Non-elective surgery |
| El Baz 2008^16^ | 6 | **Physical functioning:** Higher EurosSCORE (B=-.19, p=0.009), readmission to hospital within 6 weeks (B-0.158, p=0.0.018), higher NYHA class (B-0.150, p=0.025). P**hysical role**: readmission (B-0.217, p=0.003), longer LOS (B-0.14, p=0.046), **Bodily pain**: Smoking (B-0.168, p=0.019), history of renal insufficiency (B-0.168, p=0.025). **General physical health:** readmission to hospital (B-0.173, p=0.014). **Social role**: Readmission (B-0.225, p<0.001), LOS (B-0.160, p<0.05). **Emotional role**: Smoking (B-0.174, p<0.05). **Mental health**: Reexploration (B-0.206, p<0.01), sternal resuturing (B-0.142, p<0.05), readmission within 6 weeks (B-0.185, p<0.001), angina (B0.128, p<0.05). **Vitality**: Reexploration (B-0.170, p<0.05), Readmission (B-0.218, p<0.01). | **Physical functioning**: marital status, education, work, smoking, use of inotropes, atrial arrhythmia, ventricular arrhythmia, re-exploration, sternal resuutring, time on mechanical ventilation, LOS, angina, MI, renal diseases, diabetes, hypertension;  **Physical role**: marital status, education, work, EuroSCORE, NYHA, use of inotropes, atrial arrhythmia, ventricular arrhythmia, re-exploration, sternal resuutring, time on mechanical ventilation, angina, MI, renal diseases, diabetes, hypertension. **Bodily pain**: marital status, education, work, smoking, EuroSCORE, NYHA, use of inotropes, atrial arrhythmia, ventricular arrhythmia, re-exploration, sternal resuutring, time on mechanical ventilation, readmission, LOS, angina, MI, diabetes, hypertension. **General health**: marital status, education, work, smoking, NYHA, EuroSCORE, use of inotropes, atrial arrhythmia, ventricular arrhythmia, re-exploration, sternal resuutring, time on mechanical ventilation, LOS, angina, MI, renal diseases, diabetes, hypertension. **Social role**: marital status, education, work, smoking, NYHA, EuroSCORE, use of inotropes, atrial arrhythmia, ventricular arrhythmia, re-exploration, sternal resuturing, time on mechanical ventilation, angina, MI, renal diseases, diabetes, hypertension. **Emotional role**: marital status, education, work, NYHA, EuroSCORE, use of inotropes, atrial arrhythmia, ventricular arrhythmia, re-exploration, sternal resuutring, time on mechanical ventilation, readmission, LOS, angina, MI, renal diseases, diabetes, hypertension. **Mental health**: marital status, education, work, smoking, NYHA, EuroSCORE, use of inotropes, atrial arrhythmia, ventricular arrhythmia, time on mechanical ventilation, LOS, MI, renal diseases, diabetes, hypertension;  **Vitality:** marital status, education, work, smoking, NYHA, EuroSCORE, use of inotropes, atrial arrhythmia, ventricular arrhythmia, sternal resuturing, time on mechanical ventilation, LOS, angina, MI, renal diseases, diabetes, hypertension.  *EuroSCORE - not associated with any of 4 mental components of SF-36* |
| Jokinen 2008^17^ | 8.2 years | **Energy:** Diabetes (RR 1.03, 1.003-1.058, p<0.05), low energy score at 15 months ((RR 1.032, 1.008-1.057, p<0.05), Protective use of statin (RR 0.971 0.949-0.993, p<0.05), treatment in ICU>3days (RR 1.039, 1.008-1.070, p<0.05). **Sleep:** High pain score at 15 months (RR 1.044, 1.009-1.080, p<0.05). **Pain:** Diabetes: (RR 1.054, 1.010-1.100, p<0.05). **Emotion:** Duration of cardiac symptoms pre-op >120 days (RR 1.067, 1.007-1.131, p<0.05). **Mobility:** Diabetes: 1(RR 1.061, 1.011-1.113, p<0.05), low energy score at 15 months (RR 1.045-1.086, p<0.05). | **Social isolation** - none. **Variables NS for all NHP domains were**: sex, age (>75) at time of surgery, education, family status, pre-op NYHA I/II vs III/IV, rhythm disturbances, smoking, hypertension, hypercholesteraemia, renal failure, performed surgical procedure, 3 vessel disease, EF <50%, LMS >50%, aortic occlusion time >120mins, post-op MI, cardiac re-operation, post-op cerebral event, betablocker, calcium channel blocker, angiotensin converting enzyme inhibitor, angiotensin II agonist, diuretic, acetylsalicyclic acid, warfarin, other antithrombotic, digitalis, nitroglycerin, insulin, diabetic medication (oral), pulmonary disease medication, antidepressant, hypnotics |
| Peric 2008^18^ | 6 | NHP part 1 score for every QoL section significantly improved at 6m (p<0.001).  **Physical mobility domain**: Diabetes mellitus (8.09 (2.04-32.09), p=0.003, EF (0.73 (0.56-0.95), p=0.047), **Social Isolation domain**: Complications (4.63 (1.79-11.99), p=0.002), Emotional reaction domain: EF (0.83, (0.60-0.93), p=0.03), **Energy domain**: none,  **Sleep domain**: complications (2.71 (1.12-6.51), p=0.03), **Pain domain**: Gender (4.25 (1.74-10.47) p=0.002), complications (3.39 (1.45-7.97) p=0.005) | **Physical domain**: smoking, Hypercholesteraemia), BMI, Physical inactivity, No of grafts, Complications,  **Social isolation**: Hypercholesteraemia, Stress, complications. **Emotional reaction:** diabetes mellitus, Heredity, BMI, Stress, Comorbid diseases. **Energy domain**: age, stress, EF, **Pain domain:** Duration of CHD |
| Deaton 2009^19^ | 3 | **PCS:** CCI (1 pt increase) p=0.02, GDS (1 pt incr) p=0.003, female sex p=0.005, living alone p=0.011, BMI p=0.024, prolonged LOS (1 day increase) p<0.001. | **PCS:** Baseline PCS p=0.71, NYHA class p=0.076 |
| Herlitz 2009^20^ | 15 | **Independent predictors of inferior QoL at 15yrs** (OR (95%)CI, p): Age (per yr): 1.04 (1.02-1.06), p<0.0001; Female sex: 1.9 (1.2-3.0), p=0.007; Diabetes mellitus: 2.6 (1.2-5.7), p=0.02; Obesity: 2.2 (1.3-3.9), p=0.005; Sqrt (ICU time): 2.4 (1.1-5.6), p=0.04; Inotropic drugs: 2.8 (1.5-5.1), p=0.0009. **Independent predictors of improved (in relation to pre-op) QoL at 15yrs** (OR(95%CI), p): Pre-op inferior QoL: 3.3 (2.1-5.1, p<0.0001; Age (per yr): 0.92 (0.89-0.95), p<0.0001; Functional class (NYHA): 1.8 (1.2-2.6), p=0.0002. | Previous MI, congestive heart failure, hypertension, renal dysfunction, claudication, current smoker, previous PTCA, three vessel disease, EF <0.40, angina duration, history of valvular disease, COPD on medication, left main and proximal left anterior descending stenosis, urgency of surgery, extracorporeal circulation time, aortic cross-clamp time, number of distal anastomoses, internal mammary artery grafting, endarterectomy, time in intensive care unit, time on a ventilator, need for reoperation, neurological complication, pneumothorax, hydrothorax, supraventricular arrhythmia, need for inotropic drugs, prolonged reperfusion in heart-lung machine, need for circulatory assistance device, serum-aspartate aminotransferase maximum within 48h. |
| Maisano 2009^21^ | 2.8 ± 1.2 yrs | **Factors influencing of MLHF score:** Preop atrial fibrillation: p=0.019, Diabetes: p=0.03, Higher creatinine level: p = 0.0009, higher EuroSCORE: p = 0.02, mitral regurgitation grade at follow-up echo: p = 0.002, Systolic PAP at follow-up: p = -.04. **Factors predicting a MLHF score >30:**  Age: OR 0.11 (0.02 - 0.75), p = 0.02, Preop diabetes: OR 3.9 (1.14 - 17.16); p = 0.04, Preop NYHA class: OR 5.9 (1.4 - 29.7); p = 0.02, EF at follow-up: OR 18.7 (0.91 - 405.0), p = 0.05 | **Prediction of MLHF score:** Age at follow-up, Preoperative, NYHA class, Charlston score, Pre-operative PAPs, EF at follow-up, Sinus rhythm vs atrial fibrillation or pace maker at follow-up, LVEDD at follow-up, LVESD |
| Rantanen 2009^22^ | 6 and 12 | **6 months: *Patients (Model 1) adjusted R2=0.62*** 15D score 1 month after operation b=0.488, t=9.124, p=0.001, Physical exertion causes symptoms b=-0.073, t=-6.741, p=0.001, Symptoms on mild exertion or at rest b=-0.129, t=-5.396, p=0.001, Experienced myocardial infarction b=0.046 t=3.052 p=0.003, Other disease b=-0.031 t=-2.859 p=0.005, Aid from network members b=0.012, t=2.240, p=0.027 ***Significant Others (Model 2) adjusted R2=0.70*** 15D score at 1 month after patient’s operation b.831, t=15.168, p=0.001, Chronic disease b=-0.027, t=-2.871, p=0.005  **12 months:**  ***Patients (Model 1) adjusted R2=0.53*** 15D score 1 month after operation b=0.415, t=6.996, p<0.001, Physical exertion causes symptoms b=-0.078, t=-5884, p<0.001, Symptoms on mild exertion or at rest b=-0.094, t=-4282, p<0.001, Pre-op other disease b=-0.026 t=-2.149 p=0.033, Age b=-0.001, t=-2.013, p=0046 ***Significant Others (Model 2) adjusted R2=0.67*** 15D score at 1 month after patient’s operation b=0.755, t=14.517, p<0.001, Chronic disease b=-0.028, t=-2.809, p=0.006 | None stated |
| Juergens 2010^23^ | 3 | **Physical functioning (SF-12):** illness perception questionnaire – revised (IPQ-R) β -0.437, R2 = 0.373, p<0.05 | **Physical functioning (SF-12)** Age: β -0.005, p>0.05; Pre-surgery SF-36: β 0.189, p > 0.05; ejection fraction β=0.056, p >0.05, EuroSCORE β= -0.136, p >0.05 |
| Peric 2010^24^ | 6 | [OR (95%CI), p] **Physical mobility:** Diabetes mellitus: 4.94 (1.64-14.87), p=0.005; Complications: 3.60 (1.29-10.02), p=0.01; **Social Isolation**: Smoking: 1.86 (1.04-3.34), p=0.04; Complications: 3.77 (1.58-8.99), p=0.003; **Sleep**: Complications: 3.60 (1.51-8.58), p=0.004; **Pain:** Gender 3.93 (1.74-8.88), p=0.001; Complications: 2.43 (1.13-5.25), p=0.02. | **Physical mobility**: Marital status; smoking; hypercholesteraemia; BMI; physical inactivity; ejection fraction; EurSCORE; Associate procedure. **Social Isolation**: Marital status; hypercholesteraemia, stress, NHYA class; Associate procedure. **Emotional reaction:** Hypercholesteraemia; stress, ejection fraction, comorbid diseases. **Energy:** Age; stress; ejection fraction. **Sleep:** Gender; heredity; physical inactivity; Number of diseased vessels; CCS class; Ejection fraction; segment wall motion, associate prcedure, LITA graft. **Pain:** Duration of CHD |
| Grady 2011^25^ | Over time (0, 3, 6, 12 and 24) | **PCS:** female gender, non-white race, higher BMI, repeat cardiac operation, NHYA class, presence of CAD, heart failure, COPD, hypertension, PVD, prior MI, older age  **MCS:** Younger age, female, NHYA class, BMI, heart failure | **MCS:** gender, cardiac operation |
| Vainiola 2013^26^ | 6 | Male (coeff -0.020, p=0.016), diabetes mellitus (coeff -0.-27, p=0.004), baseline 15-D (coeff 0.531, p<0.001), severe or unbearable pain (coeff -0.023, p=0.013), restlessness during ICU treatment (coeff -0.47, p<0.001). | Age, neurological disease, respiratory disease, hypertension, hyperlipidaemia, other comorbidity, smoking, NHYA class, EuroSCORE, BMI, waiting time (days), procedure type, SOFA score, TISS score, ventilator treatment (mins), intra-aortic balloon pump, renal complication, respiratory complication, neurological complication, arrhythmia, urgent sternotomy, re-operation, nosocomial infection, deep sedation. |
| Kurfirst 2014^27^ | 12 | Pre-op PCS (OR 1.03 1.00-1.05, p=0.0187), pre-op MCS (OR 1.02, 0.997-1.00, p=0.0846) | Gender, age >70yrs, Type of surgery |
| Humphreys 2016^28^ |  | **Bodily Pain QoL Domain:** Alcohol use p=<0.05, Depression b-0.39, p=<0.013. **General Health QoL Domain:** Alcohol use p=<0.01, CHF p=<0.05, Depression b-0.33, p=0.038, Delirium b-0.22, p=0.004. **Vitality QOL Domain**Alcohol use p=<0.01, Depression b-0.32, p=<0.020. **Social functioning QoL Domain:** Alcohol use p=<0.05, Health Behaviours p=<0.05, Depression b-0.51, p=≤0.001. **Emotional Role QoL Domain:** Alcohol use p=<0.01, Cardiovascular disease p=<0.05, Depression b-0.44 p=0.003. **Mental Health QoL Domain** Alcohol use p=<0.05. **Physical Functioning QoL Domain:** Alcohol use p=<0.01, CHF p=<0.01 Hypertension p=<0.01, Aboroginal p=<0.05, Age p=<0.05. **Physical Role QoL Domain:** Alcohol use p=<0.05, Age p=<0.01 | **Bodily Pain QoL Domain:** Anxiety, stress, delirium, male, aboriginal, chronic lung disease, CHF, age, hypertension, hypercholesterolemia, diabetes, PVD, cardiovascular disease, emergent surgery LVEF, smoking, recent MI, health behaviours. **QoL Domain:** Anxiety, stress, male, aboriginal, chronic lung disease, age, hypertension, hypercholesterolemia, diabetes, PVD, cardiovascular disease, emergent surgery LVEF, smoking, recent MI, health behaviours. **Vitality QoL Domain:** Anxiety, stress, delirium, male, aboriginal, chronic lung disease, CHF, age, hypertension, hypercholesterolemia, diabetes, PVD, cardiovascular disease, emergent surgery LVEF, smoking, recent MI, health behaviours. **Social Functioning QoL Domain**  Anxiety, stress, delirium, male, aboriginal, chronic lung disease, CHF, age, hypertension, hypercholesterolemia, diabetes, PVD, cardiovascular disease, emergent surgery LVEF, smoking, recent MI. **Emotional Role QoL Domain:** Anxiety, stress, delirium, male, aboriginal, chronic lung disease, CHF, age, hypertension, hypercholesterolemia, diabetes, PVD, emergent surgery LVEF, smoking, recent MI, health behaviours. **Mental Health QoL Domain:** Depression, anxiety, stress, delirium, male, aboriginal, chronic lung disease, CHF, age, hypertension, hypercholesterolemia, diabetes, PVD, cardiovascular disease, emergent surgery LVEF, smoking, recent MI, health behaviours. **Physical Functioning QoL Domain:** Depression, anxiety, stress, delirium, male, chronic lung disease, hypercholesterolemia, diabetes, PVD, cardiovascular disease, emergent surgery LVEF, smoking, recent MI, health behaviours. **Physical Role QoL Domain:** Depression, anxiety, stress, delirium, male, aboriginal, chronic lung disease, CHF, hypertension, hypercholesterolemia, diabetes, PVD, cardiovascular disease, emergent surgery LVEF, smoking, recent MI, health behaviours |
| Patron 2016^29^ | 12 | **PCS:** pre-op PCS (var of R2 0.11, p<0.01); Education (yrs) (Var of R2 0.05, p<0.05), EuroSCORE (var R2 0.08, p<0.05), CES-D (var R2 0.30, p<0.05). **MCS**: Pre-op MCS (var of R2 0.19, p<0.001), CES-D (var of R2 0.28, p<0.05) | **PCS:** CPB duration, Instrumental activities of daily living  **MCS**: Education (yrs), CPB duration, Instrumental activities of daily living, EuroSCORE |
| Bjonnes 2017^30^ | 2 weeks, 3, 6, and 12 | **EQ 15D:** Age (coeff -0.013 [-0.026- -0.001] p=0.035); Sex and marital status (coeff -0.04 [-0.07- -0.008] p=0.015); Educational status: (secondary - coeff 0.019 [0.001-0.037] p=0.037; post-secondary - coeff 0.019 [0.002-0.038] p=0.048); Back/neck problems (coeff -0.03 [-0.04- -0.013] p<0.001); Depression (coeff -0.05 [-0.08- -0.025] p<0.001); Pain intensity (coeff -0.02 [-0.022-0.018], p<0.001). | Sex, marital status |
| Norkiene 2018^31^ | 12 | **SF36 total**: Preoperative PCS, preoperative MCS (data not stated) | Age, Gender, operation type |
| Bishawi 2018^32^ | 12 | **SAQ-QoL**: COPD (0.507 [0.276-0.932] p=0.029); Depression (0.332 [0.168-0.655] p=0.002); Baseline SAQ-QoL (0.958 [0.946-0.969] p<0.001)  **SF36 PCS**: Diabetes mellitus (0.659 [0.463-0.939] p=0.021), baseline VR36 PCS (0.918 [0.901-0.935] p < 0.001)  **SF36 MCS**: CVA (0.54 (0.325-0.898) p 0.018), depression (0.376 [0.239-0.592] p<0.001); baseline VR36 MCS (0.935 [0.920-0.948] p < 0.001) | **SAQ-QoL**: Age, Smoking, Diabetes, LVEF. **SF36 PCS**: Age, Education, Elective status, Prior CABG procedure, PVD, Depression. **SF36 MCS:** Age |
| Grand 2018^33^ | 6 | **SF36 PCS**: ASA physical status classification III (4.95 [2.30-10.63] p<0.0001); CPB duration (min) (1.01 [1.0-1.01] p = 0.038); Renal replacement for acute renal failure (24 [2.34-246] p = 0.007); Mechanical ventilation > 48 hours (6.80 [2.14-21.6] p=0.0012). **SF36 MCS**: Preoperative angina (2.16 [1.23-3.81] p=0.007); Postoperative dobutamine (2.03 [1.07-3.80] p=0.03); Renal replacement for acute renal failure (7.36 [1.21-18.9] p=0.03); Mechanical ventilation > 48 hours (6.44 [2.20-18.9] p=0.0007) | **SF36 PCS**: Age, BMI, EuroSCORE I, MDRD, active smoking, MI within 90 days, LVEF, LVEF < 30%, initial PCS score (adjustment factor), initial MCS score, Type of surgery (CABG and valve replacement), minimally invasive surgery, aortic cross-clamp duration, SOFA score, ICU, LOS > 5 days, IGSII, dobutamine, norepinephrine, IABP, ECMO, dialysis for acute renal failure, Neurologic disorders, mechanical ventilation  **SF36 MCS**: Age,BMI, EuroSCORE I,MDRD, COPD, unstable angina, LV-EF < 30%, initial PCS score, initial MCS score (adjustment factor), Type of surgery (CABG and valve replacement), CPB duration, SOFA score, ICU LOS > 5 days, GSII, ECMO, dialysis for acute renal failure, neurologic disorders, mechanical ventilation, |
| Coelho 2019^34^ | 12 | **SF36 PCS**: preoperative PCS (coeff 0.195 p< 0.001); sex (coeff 0.192 p< 0.001); age (coeff -0.18 p< 0.001); hospital LOS (coeff -0.098 p=0.039). **SF36 MCS**: Preoperative MCS (coeff 0.192 p<0.001), ICU LOS (coeff -0.126 p=0.012) | **SF36 PCS**: preoperative MCS, procedure, education level, earnings, EuroSCORE I, body mass index, NYHA and CCS, ICU LOS. **SF36 MCS**: preoperative PCS, sex, age, procedure, education level, earnings, EuroSCORE I, body mass index, NYHA and CCS, total length of hospital stay. |
| Blokzijl 2019^35^ | 10 – 14 | **SF36 PCS**: Baseline PCS (p<0.001), pulmonary disease (p<0.001), LV function (p<0.001), renal disease (p<0.001). **SF36 MCS**: Baseline MCS (p<0.001), LV function (p<0.001) | **PCS:** EF<30%, age, previous cardiac surgery; **MCS:** age, sex, EF<30% and previous cardiac surgery |
| Joskowiak 2019^36^ | 3 and 12 | **SF36 PCS**: Baseline PCS (1.08 [1.02 – 1.15] p=0.016), age (6.24 [1.22 – 31.84] p=0.028), pre-operative neurological disease (10.65 {2.04 – 55.53] p = 0.005). **SF36 MCS**: Baseline MCS (1.07 [1.03 – 1.12] p = 0.002), age (2.40 [1.05 – 5.45] p = 0.037), CABG procedure (2.56 [1.12 – 5.86] p = 0.026) | Not explicitly reported |
| Perrotti 2019^37^ | 10 years | **SF36 PCS**: diabetes (4.2 [1.67 - 8.20], p = 0.048); dyspnea (3.32 [1.47 - 6.28], p=0.002). **SF36 MCS**: Angina (2.57 [1.35 - 3.21], p = 0.018) | Demographics: sex, age, marital status, area of residence (rural vs urban), professional activity (in paid employment or not), invalidity status, socio-economic class, level of education (less than high school vs high school diploma and higher); Comorbidities: diabetes (MCS only), chronic obstructive pulmonary disease (COPD), peripheral artery disease, atrial fibrillation, anxiety and depression (HADS), physical disability, angina (assessed by CCS) (PCS only), dyspnea (NYHA class) (MCS only), LVEF, surgical complications including: periop MI, low cardiac output syndrome, mechanical ventilation support > 24 hrs, reintubation, brain injury, need for dialysis, pneumonia, sepsis, sternal wound infection, any consequent surgery required |
| Kube 2020^38^ | 6 | **SF12 – Physical QoL:** presurgical physical QoL (coeff 0.257, p <0.05), age (coeff 0.279, p < 0.05) (p*resurgery model*); post surgery QoL (coeff 0.239, p <0.05), post surgery expectations (coeff 0.348, p <0.01) (p*ost surgery model)*  **SF12 – Psychological QoL:** presurgical psychological QOL (coeff 0.369, p <0.01), presurgical expectations (coeff 0.278, p < 0.05) (p*resurgery model*); post surgical psychological QoL (coeff 0.334, p <0.01), post surgical expectations (coeff 0.301, p <0.05) (p*ost surgery model*) | Not explicitly reported |
| Rijnhart de Jong 2020^39^ | 12 | **SF36 PF:** Baseline SF 36 PF (0.954 [0.942 - 0.965], p <0.001); Diabetes (0.437 [0.265 - 0.720], p = 0.001); Female sex (0.492 [0.307 - 0.789], p=0.0030); Infection (0.240 [0.109 - 0.525], p <0.001); PCI <1 yr (0.113 [0.036 - 0.349], p <0.001) | BMI; Comorbidities: CVA, neuro dysfunction; Cardiac status: unstable angina, EuroSCORE, age, serum creatinine, extracardiac arteriopathy, poor mobility, previous cardiac surgery, chronic lung disease, active endocarditis, critical preop state, NYHA Class III or IV, Angina CCS Class IV, LVEF, Recent MI, pulmonary hypertension, urgency of surgery, number of procedures (e.g. isolated CABG, 2 procedures etc), Aortic cross-clamp time, Extra-corporeal circuit time, first extubation (hrs), resternotomy, ICU days, ICU stay extended, CVA, Readmission to ICU, readmission to hospital, perioperative MI, MI excluding perioperative MI, |
| Schaal 2020^40^ | 6 | **NHP**: Gender – male (coeff 0.39, p <0.001);  Age (>74 as reference) (<60: coeff 0.80, p <0.001; 70 – 74: coeff 0.42, p <0.001); Marital status (married as reference) - living alone (coeff 0.06, p <0.05); Occupation - no (coeff 0.13, p <0.001); Surgery – CABG (coeff 0.07, p <0.01); Chest pain (coeff 0.34, p <0.001); NYHA (I as reference) (II: coeff 0.27, p <0.001; III: coeff 0.91, p<0.001; IV: coeff 0.0.97, p<0.001); | Not explicitly stated |

Abbreviations: AF: Atrial Fibrillation; AMI: Acute Myocardial Infarction; AP: Angina Pectoris; ASA: American Society of Anesthesiologists risk classification; BMI: Body Mass Index; BP: Blood pressure; CABG: Coronary Artery Bypass Graft; CCI: Charlson Co-morbidity Index; CCS: Canadian Cardiovascular Society; CES-D: Centre for Epidemiological Study of Depression; CHD: Coronary Heart Disease; CHF: Chronic Heart Failure; COPD: Chronic Obstructive Pulmonary Disease; CPB: Cardiopulmonary Bypass; CPVD: Cerebro- or peripheral vascular disease); CVA: Cerebrovascular Accident; CVD: Cardiovascular Disease; ECC: Not stated in paper; ECMO: Extracorporeal Membrane Oxygenation; EF: Ejection Fraction; EFQ: Everyday Functioning Questionnaire; FEV1: Forced Expiratory Volume; FT: Feeling thermometer; GDS: Geriatric Depression Scale; GHS: General Health Score; HADS: Hospital Anxiety and Depression Score; IABP: Intra-Aortic Balloon Pump; ICU: Intensive Care Unit; IGS II: Indice de gravite simplifie; IMA: Internal mammary artery; IPQ-R: Illness Perception Questionnaire; ITU: Intensive Therapy Unit; LAD: Left anterior descending; LMS: Left Main Stem; LOS: Length of Stay; LVEDD: Left Ventricular End Diastolic Diameter; LVEF: Left ventricular ejection fraction; LVESD: Left Ventricular End Systolic Diameter; MCS: Mental Component Score; MDRD: Modification of Diet in Renal Disease equation for glomerular filtration rate; MI: Myocardial Infarction; MLHF: Minnesota Living with Heart Failure NHP: Nottingham Health Profile; NYHA: New York Heart Association; PAP: Pulmonary Artery Pressure; PAS: Physical Activity Score; PCI: Percutaneous Coronary Intervention; PCS: Physical Component Score; PGWBI: Psychological General Well-Being Index; POMS: Profile of Mood States; PTCA: Percutaneous Transluminal Coronary Angioplasty; PVD: Peripheral Vascular Disease; QoL: Quality of Life; QoR: Quality of Recovery; S-ASAT: Aspartate Aminotransferase; SAQ: Seattle Angina Questionnaire; SOFA: Sequential Organ Failure Score; SVT: Supraventricular tachycardia; TIA: Transient Ischaemic Attack; TISS: Therapeutic Intervention Scoring System; VR-36: Rand 36 (version of SF26); Yr: year

**REFERENCES**

1. Myles PS, Hunt JO, Fletcher H, et al. Relation between Quality of Recovery in Hospital and Quality of Life at 3 Months after Cardiac Surgery. *Anesthesiology* 2001; 95: 862–867.

2. Baldassarre FG, Arthur HM, DiCenso A, et al. Effect of coronary artery bypass graft surgery on older women’s health-related quality of life. *Hear Lung J Acute Crit Care* 2002; 31: 421–431.

3. Falcoz PE, Chocron S, Stoica L, et al. Open Heart Surgery: One-Year Self-Assessment of Quality of Life and Functional Outcome. *Ann Thorac Surg* 2003; 76: 1598–1604.

4. Herlitz J, Brandrup-Wognsen G, Caidahl K, et al. Improvement and factors associated with improvement in quality of life during 10 years after coronary artery bypass drafting. *Coron Artery Dis* 2003; 14: 509–517.

5. Schelling G, Richter M, Roozendaal B, et al. Exposure to high stress in the intensive care unit may have negative effects on health-related quality-of-life outcomes after cardiac surgery. *Crit Care Med* 2003; 31: 1971–1980.

6. Baberg HT, Dirlich M, Laczkovics A, et al. Determinants of health-related quality of life after aortic valve replacement in six-month survivors of intervention. *J Heart Valve Dis* 2004; 13: 914–920.

7. Jarvinen O, Julkunen J, Saarinen T, et al. Perioperative myocardial infarction has negative impact on health-related quality of life following coronary artery bypass graft surgery. *Eur J Cardio-Thoracic Surg* 2004; 26: 621–627.

8. Rumsfeld JS, Ho PM, Magid DJ, et al. Predictors of health-related quality of life after coronary artery bypass surgery. *Ann Thorac Surg* 2004; 77: 1508–1513.

9. Al-Ruzzeh S, Athanasiou T, Mangoush O, et al. Predictors of poor mid-term health related quality of life after primary isolated coronary artery bypass grafting surgery. *Heart* 2005; 91: 1557–62.

10. Herlitz J, Brandrup-Wognsen G, Caidahl K, et al. Determinants for an impaired quality of life 10 years after coronary artery bypass surgery. *Int J Cardiol* 2005; 98: 447–452.

11. Le Grande MR, Elliott PC, Murphy BM, et al. Health related quality of life trajectories and predictors following coronary artery bypass surgery. *Health Qual Life Outcomes* 2006; 4: 49.

12. Myles PS, Viira D, Hunt JO. Quality of life at three years after cardiac surgery: relationship with preoperative status and quality of recovery. *Anaesth Intensive Care* 2006; 34: 176–83.

13. Noyez L, Markou A, van Breugel F. Quality of life one year after myocardial revascularization. Is preoperative quality of life important? *Interact Cardiovasc Thorac Surg* 2006; 5: 115–120.

14. Panagopoulou E, Montgomery A, Benos A. Quality of life after coronary artery bypass grafting: evaluating the influence of preoperative physical and psychosocial functioning. *J Psychosom Res* 2006; 60: 639–644.

15. Dunning J, Waller JRL, Smith B, et al. Coronary Artery Bypass Grafting is Associated With Excellent Long-Term Survival and Quality of Life: A Prospective Cohort Study. *Ann Thorac Surg* 2008; 85: 1988–1993.

16. El Baz N, Middel B, Van Dijk JP, et al. EuroSCORE predicts poor health-related physical functioning six month postcoronary artery bypass graft surgery. *J Cardiovasc Surg (Torino)* 2008; 49: 663–72.

17. Jokinen JJ, Hippelainen MJ, Hanninen T, et al. Prospective assessment of quality of life of octogenarians after cardiac surgery: factors predicting long-term outcome. *Interact Cardiovasc Thorac Surg* 2008; 7: 813–818.

18. Peric V, Borzanovic M, Stolic R, et al. Predictors of Worsening of Patients’ Quality of Life Six Months After Coronary Artery Bypass Surgery. *J Card Surg* 2008; 23: 648–654.

19. Deaton C, Thourani V. Patients with Type 2 Diabetes Undergoing Coronary Artery Bypass Graft Surgery: Predictors of Outcomes. *Eur J Cardiovasc Nurs* 2009; 8: 48–56.

20. Herlitz J, Brandrup-Wognsen G, Evander MH, et al. Quality of life 15 years after coronary artery bypass grafting. *Coron Artery Dis* 2009; 20: 363–369.

21. Maisano F, Viganò G, Calabrese C, et al. Quality of life of elderly patients following valve surgery for chronic organic mitral regurgitation. *Eur J Cardio-thoracic Surg* 2009; 36: 261–266.

22. Rantanen A, Kaunonen M, Tarkka M, et al. Patients’ and significant others’ health-related quality of life one month after coronary artery bypass grafting predicts later health-related quality of life. *Hear Lung J Acute Crit Care* 2009; 38: 318–329.

23. Juergens MC, Seekatz B, Moosdorf RG, et al. Illness beliefs before cardiac surgery predict disability, quality of life, and depression 3 months later. *J Psychosom Res* 2010; 68: 553–560.

24. Peric V, Borzanovic M, Stolic R, et al. Quality of life in patients related to gender differences before and after coronary artery bypass surgery☆. *Interact Cardiovasc Thorac Surg* 2010; 10: 232–238.

25. Grady KL, Lee R, Subačius H, et al. Improvements in Health-Related Quality of Life Before and After Isolated Cardiac Operations. *Ann Thorac Surg* 2011; 91: 777–783.

26. Vainiola T, Roine RP, Suojaranta-Ylinen R, et al. Can factors related to mortality be used to predict the follow-up health-related quality of life (HRQoL) in cardiac surgery patients? *Intensive Crit Care Nurs* 2013; 29: 337–43.

27. Kurfirst V, Mokráček A, Krupauerová M, et al. Health-related quality of life after cardiac surgery--the effects of age, preoperative conditions and postoperative complications. *J Cardiothorac Surg* 2014; 9: 46.

28. Humphreys JM, Denson LA, Baker RA, et al. The importance of depression and alcohol use in coronary artery bypass graft surgery patients: Risk factors for delirium and poorer quality of life. *J Geriatr Cardiol* 2016; 13: 51–57.

29. Patron E, Messerotti Benvenuti S, Palomba D. Preoperative biomedical risk and depressive symptoms are differently associated with reduced health-related quality of life in patients 1 year after cardiac surgery. *Gen Hosp Psychiatry* 2016; 40: 47–54.

30. Bjørnnes AK, Parry M, Falk R, et al. Impact of marital status and comorbid disorders on health-related quality of life after cardiac surgery. *Qual Life Res* 2017; 26: 2421–2434.

31. Norkienė I, Urbanaviciute I, Kezyte G, et al. Impact of pre-operative health-related quality of life on outcomes after heart surgery. *ANZ J Surg* 2018; 88: 332–336.

32. Bishawi M, Hattler B, Almassi GH, et al. Preoperative factors associated with worsening in health-related quality of life following coronary artery bypass grafting in the Randomized On/Off Bypass (ROOBY) trial. *Am Heart J* 2018; 198: 33–38.

33. Grand N, Bouchet JB, Zufferey P, et al. Quality of Life After Cardiac Operations Based on the Minimal Clinically Important Difference Concept. *Ann Thorac Surg* 2018; 106: 548–554.

34. Coelho PNMP, Miranda LMRPC, Barros PMP, et al. Quality of life after elective cardiac surgery in elderly patients. *Interact Cardiovasc Thorac Surg* 2019; 28: 199–205.

35. Blokzijl F, Houterman S, Van Straten BHM, et al. Quality of life after coronary bypass: A multicentre study of routinely collected health data in the Netherlands. *Eur J Cardio-thoracic Surg* 2019; 56: 526–533.

36. Joskowiak D, Meusel D, Kamla C, et al. Impact of Preoperative Functional Status on Quality of Life after Cardiac Surgery. *Thorac Cardiovasc Surg*. Epub ahead of print 2019. DOI: 10.1055/s-0039-1696953.

37. Perrotti A, Ecamot F, Monaco F, et al. Quality of life 10 years after cardiac surgery in adults: a long-term follow-up study. *Health Qual Life Outcomes* 2020; 18: 1–10.

38. Kube T, Meyer J, Grieshaber P, et al. Patients’ pre- and postoperative expectations as predictors of clinical outcomes six months after cardiac surgery. *Psychol Heal Med* 2020; 25: 781–792.

39. Rijnhart-De Jong H, Haenen J, Bol Raap G, et al. Determinants of non-recovery in physical health-related quality of life one year after cardiac surgery: A prospective single Centre observational study. *J Cardiothorac Surg* 2020; 15: 1–10.

40. Schaal NK, Assmann A, Rosendahl J, et al. Health-related quality of life after heart surgery – Identification of high-risk patients: A cohort study. *Int J Surg* 2020; 76: 171–177.
